# Hepatitis C in Children—An Asia–Pacific Concise Perspective

**DOI:** 10.3390/pathogens13100860

**Published:** 2024-10-01

**Authors:** Lynette Goh, Winita Hardikar

**Affiliations:** 1 Department of Gastroenterology, Hepatology and Nutrition, KK Women’s and Children’s Hospital, Singapore 229899, Singapore; 2 Gastroenterology and Clinical Nutrition, The Royal Children’s Hospital Melbourne, Parkville, VIC 3052, Australia

**Keywords:** hepatitis C, direct-acting antivirals, Asia–Pacific

## Abstract

Since the discovery of hepatitis C virus (HCV) in 1989, we now have curative treatment options with direct-acting antiviral therapies. By increasing the rate of treatment and reducing transmission, the eradication of HCV is potentially achievable. Nonetheless, the feasibility and implementation of this goal remains challenging. This article sums up the approach to managing children with HCV in the Asia–Pacific region and lists some of the difficulties and complexities surrounding this issue.

## 1. Introduction

In 2016, the World Health Organization (WHO) announced plans to eradicate hepatitis C virus (HCV) as a public health threat globally by 2030 [1]. With the advent of safe and efficacious direct-acting antiviral (DAA) treatments suitable for paediatric patients aged 3 years and above, it seems that we may be getting closer to this goal. However, there is currently no systematic data collection or reporting of the coverage of testing and treatment among children and adolescents [2]. This is particularly lacking in the Asia–Pacific region, where almost half of HCV-infected children live [3]. In this review, we summarize the burden of HCV in the Asia–Pacific region, the currently available treatment options, screening strategies and barriers to accessing treatment.

## 2. Epidemiology of HCV in Asia–Pacific

HCV is a single-stranded RNA virus that is categorized into six distinct genotypes, with the predominant genotypes in Asia–Pacific being 1 and 3 [4]. It has been estimated [3] that 3.26 million children and adolescents in the world are living with HCV, with Pakistan, China, India, and Nigeria alone accounting for >50% of these. Globally, the prevalence of HCV in children from 0 to 18 years old [3] has been reported to be 0.13%. Amongst the Asia–Pacific countries, this ranges from as low as 0.01% in Japan to as high as 1.02% in Pakistan [3]. Across the world, there is a higher proportion of children with HCV in countries with low and medium incomes compared to countries with high incomes [4]. Regardless, the majority subgroup comprises adolescents 12–18 years old, likely driven by injection drug use as the main mode of transmission [5]. These high HCV rates in young adults translate to an increased infection rate in young women and contributes to the vicious cycle of vertical transmission.

## 3. Natural Progression of HCV in Children

Chronic HCV in children is largely benign, taking >20 years to develop cirrhosis and hepatocellular carcinoma (HCC) [6,7]. The prevalence of advanced liver disease from chronic HCV in children is reported to be 1–2% [8]. This makes case finding in children challenging as they are mostly asymptomatic. Even though the morbidity and mortality rates in children are low, the risk of severe disease in adulthood persists [9,10].

## 4. Timing of Treatment

With DAAs showing a sustained virologic response 12 weeks after treatment completion (SVR12) of >95%, there has been a paradigm shift towards DAA therapy during childhood. A strategy of initiating DAA therapy for all children older than three years of age with HCV infection, irrespective of liver function tests, genotype, or degree of liver injury, has been adopted [11]. Now, with pan-genotypic combination regimens approved for children as young as 3 years old, subtype testing is no longer required, making it easier to initiate treatment [12].

It has been shown that treating children at 3 years of age is more cost effective per quality-adjusted life-year (QALY) gained compared to deferring treatment to 18 years of age. In addition, delaying treatment until 18 years of age results in an increased lifetime risk of late-stage liver complications, as well as increased costs of treatment [13]. According to data modelling, treating 10,000 children early would prevent 330 cases of cirrhosis, 18 cases of hepatocellular carcinoma, and 48 liver-related deaths in the next 20 years [14].

HCV impacts not only the child’s health-related quality of life [15] but also their caregivers [16]. Studies have demonstrated the significant effects on psychosocial health and cognitive functioning in children with HCV as well as their families [17], with immediate reversal shown after treatment with DAAs [18]. Furthermore, in those diagnosed <4 years of age, the accrued lifetime cost of chronic infection has been estimated to be USD 116,540 to USD 147,130 which far exceeds the cost of likely curative treatment with DAAs [19].

## 5. Treatment Regimes

There are different classes of DAAs based on their mechanism of action and therapeutic targets, namely non-structural proteins 3/4A (NS3/4A), protease inhibitors (PIs), NS5B nucleoside polymerase inhibitors (NIs), NS5B non-nucleoside polymerase inhibitors (NNIs) and NS5A inhibitors. Table 1 shows the DAAs that have been approved by both the European Medicines Agency (EMA) as well as the United States Food and Drug Administration (US FDA) for use in children ≥3 years old.

The latest European Society for Paediatric Gastroenterology Hepatology and Nutrition (ESPGHAN) recommendations published in 2024 [11] recommend the use of pan-genotypic, ribavirin-free regimens with the shortest treatment duration possible (if available). Moreover, patients should undergo swallowability assessments prior to starting treatment due to concerns of noncompliance to treatment. Regimens contain a combination of two different classes of DAAs to prevent resistance. Table 2 illustrates the currently available regimens for treatment, with the duration of the shortest course lasting 8 weeks.

**Figure 1:**
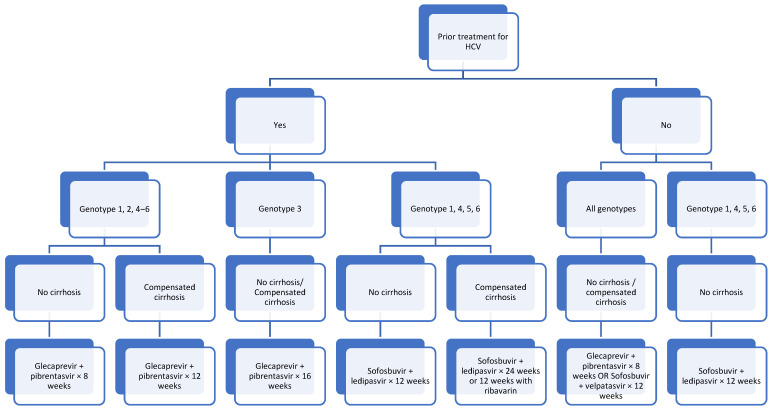
Approach to patients ≥3 years old with HCV.

In summary, therapy with DAAs is indicated for all children (≥3 years) with HCV infection, even if they are asymptomatic or have normal liver function tests; genotyping and liver biopsy are not necessary for starting treatment. Figure 1 shows the recommended approach to managing patients with HCV.

As per Figure 1, for treatment-naïve patients, considerations would be with regards to the availability and cost of treatment as well as adherence to therapy, compliance and the duration of treatment. On the other hand, for patients who have failed previous medications, genotyping is required in order to determine the treatment to proceed with. Coinfection with hepatitis B virus needs to be excluded prior to treatment.

### Additional Drugs to Consider for Patients >12 Years Old

Elbasvir + grazoprevir has been approved by EMA and FDA for patients >12 years old with genotypes 1 or 4 and can be used in the presence of compensated cirrhosis as well as no cirrhosis in the setting of both treatment-naïve patients and previous treatment failure. Elbasvir is an NS5A inhibitor and grazoprevir is an NS3/4A protease inhibitor [29]. The standard recommended dose for patients ≥30 kg is 50 mg elbasvir + 100 mg grazoprevir once a day for 12 weeks. However, those with genotype 1a infection, baseline NS5A polymorphism or those who are treatment-experienced with genotype 4 infection should be treated for 16 weeks with ribavirin. Side effects include headache (7%), fatigue (7%), and nausea (4%) [29].

## 6. Follow-Up after Treatment

It would be ideal to meet with the patient and caregivers midway through the treatment course to assess as well as reinforce compliance to medications. Blood tests to check for the clearance of HCV could be then performed 12 weeks after the completion of treatment. After achieving a sustained virological response, patients with normal liver function tests and without cirrhosis do not require additional follow-up [30]. Patients who continue to use injectable drugs or have other ongoing risk factors should undergo routine checks for HCV infection annually. In patients who have been successfully treated for HCV infection, serology cannot be used to test for re-infection as patients remain seropositive following successful treatment.

## 7. Barriers to Care

Despite being curative, cost remains a key barrier to access for many patients as DAAs are expensive drugs [31] and not all countries have a healthcare system that is able to cover or subsidize costs. An option would be for countries to manufacture their own generic versions of HCV therapies [32]. Making DAAs available at an affordable price is essential to eliminate HCV in the Asia–Pacific region. A recent publication in Lancet showed the discrepancies in the registration and reimbursement of DAAs, where countries with lower rates of HCV have greater access and subsidies available for treatment [33]. This calls for more studies looking at DAA access for children, who remain mostly absent from national HCV plans and strategies. International organizations and governments need to allocate funding to support these initiatives, ensuring that even the most resource-limited countries can effectively manage and treat hepatitis C in children.

In India, generic versions of three DAAs, namely sofosbuvir, ledipasvir and daclatasvir, are being manufactured at lower costs [34]. These have shown to be effective in the treatment of adolescents with thalassemia major and chronic HCV [35]. The use of generic DAAs is not only cost-effective but also cost-saving, where the cost of treatment is offset by the savings in future healthcare costs. Through the prevention of decompensated cirrhosis and hepatocellular carcinoma, it has been calculated that the government upfront spending budget on DAAs will start seeing a recoup on investment within 2 years of the initiation of DAA treatment.

Barber et al. has tried to estimate cost-based generic prices using an established algorithm, which accounts for costs of the active pharmaceutical ingredient (API), excipients, conversion costs of API to the finished pharmaceutical product, taxes assuming manufacture in India, and a 10% profit margin. Using this, estimated cost-based prices for a course of treatment were USD 58 for sofosbuvir/ledipasvir and USD 85 for sofosbuvir/velpatasvir [36].

## 8. Screening and Testing Practices

In line with the WHO targets to diagnose 90% of people with HCV [37], robust and comprehensive screening and testing practices need to be implemented. The currently accepted standard testing for perinatally exposed children is anti-HCV testing at age 18 months or older. The main problem with testing at 18 months of age is failure to attend, with attendance rates of only 50% [38]. In a recent analysis by Hall et al. [39], it has been shown that a more optimal timing is a single HCV RNA test during age 2–6 months. This reduces the risks of loss to follow-up, as testing further from birth may inadvertently lead to lapses over time. With the latest advancements in analytical sensitivity allowing the detection of low-level viremia, RNA testing for perinatally acquired HCV infection in early infancy has shown excellent diagnostic results [40]. Unfortunately, HCV RNA testing is scarcely available in low- and middle-income countries due to prohibitive costs [41].

The identification of cases is the first step in delivering care, but there is also a current lack of policies that outline case finding strategies and their subsequent linkage to medical care, counselling, treatment and follow-up. Regional collaboration to develop and implement policies for the prevention, screening, and treatment of hepatitis C in children is crucial; this includes the sharing of best practices and pooling of resources. The implementation of universal testing is lacking not only in the Asia–Pacific region, but also in Western countries.

## 9. Blood Banks

The transmission of HCV through contaminated blood products is estimated to be 24% [42]. In developed countries, with the use of NAT-based (nucleic acid technology) screening in blood banks, the risk of transfusion transmitted infection is close to zero, whereas in countries such as India, individual donation (ID) NAT testing is not yet compulsory, and as a result of this, HCV is still widespread amongst children who have received multiple blood transfusions. In India, the prevalence of HCV in haemophilia and thalassemia patients has been reported to be as high as 51% [43].

## 10. Universal Screening of Pregnant Women

Perinatal–vertical transmission has been found to account for up to 11% of HCV cases in children [4]. Both the American Association for the Study of Liver Diseases (AASLD) [11] and European Association for the Study of the Liver EASL [44] recommend screening all pregnant women for HCV, and this has been found to be highly cost-effective [45]. Although the Western Pacific region has largely adopted universal HCV screening for pregnant women, this is still lacking in Southeast Asia [46]. Currently, more data are emerging regarding the use of DAAs in pregnancy, and we are awaiting recommendations of a safe and effective DAA combination for these women.

## 11. Horizonal Transmission

Injection drug use amongst adolescents is increasingly prevalent, and the transmission of HCV through the sharing of needles during intravenous drug administration has been documented to be as high as 53% [4]. This subpopulation would benefit from a targeted programme as part of micro-elimination, where national elimination goals are broken down into smaller goals for specific population subgroups [47], allowing for the tackling of specific challenges such as stigmatization and marginalization. Other known risk factors for HCV transmission include tattooing and home circumcisions with published data from Turkey [48]; however, data from the Asia–Pacific region regarding this remain scarce [49]. The engagement of these subgroups to advocate for the safe use of needles as well as testing and treating them would require concerted campaigns and community-based screening.

## 12. Public Education

At the recent 33rd annual meeting of the Asian Pacific Association for the Study of the Liver (APASL), the commitment to the eradication of Hepatitis C was reiterated [50]. Representatives across the Asia–Pacific signed and confirmed their commitment to the call for action to raising awareness of the threat of viral hepatitis and the social, health, and economic benefits of hepatitis elimination. Strategies to prevent transmission include improving infection control practices in healthcare settings, ensuring safe blood transfusions, and providing education on avoiding needle-sharing and unsafe sexual practices.

## 13. Point-of-Care Testing

The FDA has recently approved the use of Cepheid’s Xpert^®^ HCV (Cepheid, Sunnyvale, CA, USA), which provides results within an hour on a small amount of blood drawn via a fingerprick. Rather than requiring a sample to be sent to a central lab for testing, the test detects HCV RNA and streamlines the management algorithm as patients can be started on treatment on the same visit within the same day. This reduces the possibility of patients defaulting follow up and is beneficial for children who may be fearful of blood-taking.

## 14. Conclusions

Addressing hepatitis C in children in the Asia–Pacific region requires a multifaceted approach that combines improved screening and testing, affordable treatment, public education, and regional cooperation. Increasing awareness and access to DAAs for children and adolescents in addition to medical subsidies for testing and treatment would require an integrated response from key stakeholders. Given this is a global health problem, and most of the infections are in the Asia–Pacific region, eradication will require a greater focus on this region.

## Figures and Tables

**Table 1:** HCV DAAs and mechanism of action.

Drug	Mechanism of Action
Sofosbuvir [20]	NS5B nucleoside polymerase inhibitor, metabolized to the active antiviral agent GS-461203. GS-461203 serves as a defective substrate for the NS5B protein, which is the viral RNA polymerase, thus inhibiting viral RNA synthesis.
Ledipasvir [21], velpatasvir [22] and pibrentasvir [23]	Inhibits NS5A protein required for viral RNA replication and the assembly of HCV virions.
Glecaprevir [24]	Inhibits the NS3/4A protease activity of cleaving the HCV polypeptide and the proteolytic processing of mature structural proteins, disrupting the viral life cycle.

**Table 2:** DAA regimens approved for children ≥3 years old.

Drug Combination	Granules/Pellets Formulation	Dose	Common Side Effects
Glecaprevir with pibrentasvir [25,26]	Yes	Once-daily dose <20 kg 150 mg glecaprevir with 60 mg pibrentasvir ≥20 kg to <30 kg 200 mg glecaprevir with 80 mg pibrentasvir ≥30 kg to <45 kg 250 mg glecaprevir with 100 mg pibrentasvir ≥45 kg or ≥12 years old 300 mg glecaprevir with 120 mg pibrentasvir	Nasopharyngitis (26%), upper respiratory tract infection (19%), headache (14–17%)
Sofosbuvir with velpatasvir [27]	Yes	Once-daily dose <17 kg 150 mg sofosbuvir with 37.5 mg velpatasvir 17 to <30 kg 200 mg sofosbuvir with 50 mg velpatasvir ≥30 kg 400 mg sofoabuvir with 100 mg velpatasvir	Headache (20%), fatigue (17%), vomiting (15%), cough (12%), nausea (10%)
Sofosbuvir with ledipasvir [28]	No	Once-daily dose >17 kg 150 mg sofosbuvir with 33.75 mg ledipasvir 17 to <35 kg 200 mg sofosbuvir with 45 mg ledipasvir ≥35 kg 400 mg sofosbuvir with 90 mg ledipasvir	Headache (18–27%), fever (17–21%), vomiting (24%)

## References

[1] WHO (2016). Global Health Sector Strategies Viral Hepatitis 2016–2021.

[2] Malik F., Bailey H., Chan P., Collins I.J., Mozalevskis A., Thorne C., Easterbrook P. (2021). Where are the children in national hepatitis C policies? A global review of national strategic plans and guidelines. JHEP Rep..

[3] Schmelzer J., Dugan E., Blach S., Coleman S., Cai Z., DePaola M., Estes C., Gamkrelidze I., Jerabek K., Ma S. (2020). Global prevalence of hepatitis C virus in children in 2018: A modelling study. Lancet Gastroenterol. Hepatol..

[4] Modin L., Arshad A., Wilkes B., Benselin J., Lloyd C., Irving W.L., Kelly D.A. (2019). Epidemiology and natural history of hepatitis C virus among children and young people. J. Hepatol..

[5] El-Sayed M.H., Razavi H. (2015). Global estimate of HCV infection in the pediatric and adolescent population. J. Hepatol..

[6] El-Shabrawi M.H., Kamal N.M. (2013). Burden of pediatric hepatitis C. World J. Gastroenterol..

[7] Kiyosawa K., Tanaka E., Sodeyama T., Furuta K., Usuda S., Yousuf M., Furuta S. (1990). Transition of antibody to hepatitis C virus from chronic hepatitis to hepatocellular carcinoma. Jpn. J. Cancer Res..

[8] Bortolotti F., Verucchi G., Cammà C., Cabibbo G., Zancan L., Indolfi G., Giacchino R., Marcellini M., Marazzi M.G., Barbera C. (2008). Long-term course of chronic hepatitis C in children: From viral clearance to end-stage liver disease. Gastroenterology..

[9] Jara P., Resti M., Hierro L., Giacchino R., Barbera C., Zancan L., Crivellaro C., Sokal E., Azzari C., Guido M. (2003). Chronic hepatitis C virus infection in childhood: Clinical patterns and evolution in 224 white children. Clin. Infect. Dis..

[10] Stinco M., Bartolini E., Veronese P., Rubino C., Moriondo M., Ricci S., Trapani S., Azzari C., Resti M., Indolfi G. (2022). Epidemiology and natural history of Childhood-Acquired chronic hepatitis C: A Single-Center Long-Term prospective study. J. Ped Gastroenterol. Nutr..

[11] Bhattacharya D., Aronsohn A., Price J., Lo Re V., AASLD-IDSA HCV Guidance Panel (2023). Hepatitis C Guidance 2023 Update: AASLD-IDSA Recommendations for Testing, Managing, and Treating Hepatitis C Virus Infection. Clin. Infect. Dis..

[12] Indolfi G., Gonzalez-Peralta R.P., Jonas M.M., Sayed M.H.E., Fischler B., Sokal E., Wirth S., Nicastro E., Kohlmaier B., Hepatology Committee of the ESPGHAN (2024). ESPGHAN recommendations on treatment of chronic hepatitis C virus infection in adolescents and children including those living in resource-limited settings. J. Pediatr. Gastroenterol. Nutr..

[13] Greenaway E., Haines A., Ling S.C., Krahn M. (2021). Treatment of Chronic Hepatitis C in Young Children Reduces Adverse Outcomes and Is Cost-Effective Compared with Deferring Treatment to Adulthood. J. Pediatr..

[14] Nguyen J., Barritt AS 4th Jhaveri R. (2019). Cost Effectiveness of Early Treatment with Direct-Acting Antiviral Therapy in Adolescent Patients with Hepatitis C Virus Infection. J. Pediatr..

[15] Nydegger A., Srivastava A., Wake M., Smith A.L., Hardikar W. (2008). Health-related quality of life in children with hepatitis C acquired in the first year of life. J. Gastroenterol. Hepatol..

[16] Rodrigue J.R., Balistreri W., Haber B., Jonas M.M., Mohan P., Molleston J.P., Murray K.F., Narkewicz M.R., Rosenthal P., Smith L.J. (2009). Impact of hepatitis C virus infection on children and their caregivers: Quality of life, cognitive, and emotional outcomes. J. Pediatr. Gastroenterol. Nutr..

[17] Rodriguez-Baez N. (2021). Hepatitis C in Young Children: To Treat or Not to Treat—Is It Cost-Effective?. J. Pediatr..

[18] Razavi H., ElKhoury A.C., Elbasha E., Estes C., Pasini K., Poynard T., Kumar R. (2013). Chronic hepatitis C virus (HCV) disease burden and cost in the United States. Hepatology.

[19] Younossi Z.M., Stepanova M., Balistreri W., Schwarz K., Murray K.F., Rosenthal P., Bansal S., Hunt S. (2018). Health-related Quality of Life in Adolescent Patients With Hepatitis C Genotype 1 Treated With Sofosbuvir and Ledipasvir. J. Pediatr. Gastroenterol. Nutr..

[20] Bhatia H.K., Singh H., Grewal N., Natt N.K. (2014). Sofosbuvir: A novel treatment option for chronic hepatitis C infection. J. Pharmacol. Pharmacother..

[21] PubChem (2004). PubChem Compound Summary for CID 67505836, Ledipasvir.

[22] Mogalian E., German P., Kearney B.P., Yang C.Y., Brainard D., Link J., McNally J., Han L., Ling J., Mathias A. (2017). Preclinical Pharmacokinetics and First-in-Human Pharmacokinetics, Safety, and Tolerability of Velpatasvir, a Pangenotypic Hepatitis C Virus NS5A Inhibitor, in Healthy Subjects. Antimicrob. Agents Chemother..

[23] PubChem (2004). PubChem Compound Summary for CID 58031952, Pibrentasvir.

[24] Salam K.A., Akimitsu N. (2013). Hepatitis C virus NS3 inhibitors: Current and future perspectives. Biomed. Res. Int..

[25] Jonas M.M., Squires R.H., Rhee S.M., Lin C.W., Bessho K., Feiterna-Sperling C., Hierro L., Kelly D., Ling S.C., Strokova T. (2020). Pharmacokinetics, safety, and efficacy of glecaprevir/pibrentasvir in adolescents with chronic hepatitis C virus: Part 1 of the DORA Study. Hepatology.

[26] Jonas M.M., Rhee S., Kelly D.A., Del Valle-Segarra A., Feiterna-Sperling C., Gilmour S., Gonzalez-Peralta R.P., Hierro L., Leung D.H., Ling S.C. (2021). Pharmacokinetics, safety, and efficacy of glecaprevir/pibrentasvir in children with chronic HCV: Part 2 of the DORA Study. Hepatology.

[27] Jonas M.M., Romero R., Rosenthal P., Lin C.H., Verucchi G., Wen J., Balistreri W.F., Whitworth S., Bansal S., Leung D.H. (2024). Sofosbuvir-velpatasvir in children 3-17 years old with hepatitis C virus infection. J. Pediatr. Gastroenterol. Nutr..

[28] Schwarz K.B., Rosenthal P., Murray K.F., Honegger J.R., Hardikar W., Hague R., Mittal N., Massetto B., Brainard D.M., Hsueh C. (2020). Ledipasvir-sofosbuvir for 12 weeks in children 3 to <6 years old with chronic hepatitis C. Hepatology.

[29] Gonzalez-Peralta R.P., Wirth S., Squires R.H., Mutschler F., Lang T., Pawlowska M., Sluzewski W., Majda-Stanislawska E., Fischler B., Balistreri W.F. (2023). Elbasvir/grazoprevir in children aged 3–18 years with chronic HCV genotype 1 or 4 infection: A pharmacokinetic modelling study. Hepatol. Commun..

[30] Hepatitis C., Virus Infection Consensus Statement Working Group (2022). Australian Recommendations for the Management of Hepatitis C Virus Infection: A Consensus Statement (2022).

[31] Rein D.B., Wittenborn J.S., Smith B.D., Liffmann D.K., Ward J.W. (2015). The cost-effectiveness, health benefits, and financial costs of new antiviral treatments for hepatitis C virus. Clin. Infect. Dis..

[32] Abdulla M., Al Ghareeb A.M., Husain H.A.H.Y., Mohammed N., Al Qamish J. (2022). Effectiveness and safety of generic and brand direct acting antivirals for treatment of chronic hepatitis C. World J. Clin. Cases.

[33] Marshall A.D., Willing A.R., Kairouz A., Cunningham E.B., Wheeler A., O’Brien N., Perera V., Ward J.W., Hiebert L., Degenhardt L. (2024). Global HCV and HIV Treatment Restrictions Group. Direct-acting antiviral therapies for hepatitis C infection: Global registration, reimbursement, and restrictions. Lancet Gastroenterol. Hepatol..

[34] Aggarwal R., Chen Q., Goel A., Seguy N., Pendse R., Ayer T., Chhatwal J. (2017). Cost-effectiveness of hepatitis C treatment using generic direct-acting antivirals available in India. PLoS ONE.

[35] Nagral A., Jhaveri A., Sawant S., Parikh N.S., Nagral N., Merchant R., Gandhi M. (2019). Treatment of Chronic Hepatitis C Infection with Direct Acting Antivirals in Adolescents with Thalassemia Major. Indian. J. Pediatr..

[36] Barber M.J., Gotham D., Khwairakpam G., Hill A. (2020). Price of a hepatitis C cure: Cost of production and current prices for direct-acting antivirals in 50 countries. J. Virus Erad..

[37] WHO (2022). Consolidated Guidelines on HIV, Viral Hepatitis and STI Prevention, Diagnosis, Treatment and Care for Key Populations.

[38] Lopata S.M., McNeer E., Dudley J.A., Wester C., Cooper W.O., Carlucci J.G., Espinosa C.M., Dupont W., Patrick S.W. (2020). Hepatitis C testing among perinatally exposed infants. Pediatrics.

[39] Hall E.W., Panagiotakopoulos L., Wester C., Nelson N., Sandul A.L. (2023). Cost-Effectiveness of Strategies to Identify Children with Perinatally Acquired Hepatitis C Infection. J. Pediatr..

[40] Gowda C., Smith S., Crim L., Moyer K., Sanchez P.K., Honegger J.R. (2020). Nucleic Acid Testing for Diagnosis of Perinatally-Acquired Hepatitis C Virus Infection in Early Infancy. Clin. Infect. Dis..

[41] WHO (2021). Accelerating Access to Hepatitis C diagnostics and Treatment. Overcoming Barriers in Low- and Middle-Income Countries.

[42] Riaz M., Abbas M., Rasool G., Baig I.S., Mahmood Z., Munir N., Mahmood Tahir I., Ali Shah S.M., Akram M. (2022). Prevalence of transfusion transmitted infections in multiple blood transfusion-dependent thalassemic patients in Asia: A systemic review. Int. J. Immunopathol. Pharmacol..

[43] Mishra K., Shah A., Patel K., Ghosh K., Bharadva S. (2020). Seroprevalence of HBV, HCV and HIV-1 and correlation with molecular markers among multi-transfused thalassemia patients in Western India. Mediterr. J. Hematol. Infect. Dis..

[44] Pawlotsky J.M., Negro F., Aghemo A., Berenguer M., Dalgard O., Dusheiko G., Marra F., Puoti M., Wedemeyer H., European Association for the Study of the Liver (2020). EASL recommendations on treatment of hepatitis C: Final update of the series. J. Hepatol..

[45] Saab S., Kullar R., Khalil H., Gounder P. (2021). Cost-effectiveness of Universal Hepatitis C Screening in Pregnant Women: A Systematic Review. J. Clin. Gastroenterol..

[46] https://www.globalhep.org/.

[47] Lazarus J.V., Wiktor S., Colombo M., Thursz M. (2017). EASL International Liver Foundation. Micro-elimination—A path to global elimination of hepatitis C. J. Hepatol..

[48] Karaca C., Cakaloğlu Y., Demir K., Ozdil S., Kaymakoğlu S., Badur S., Okten A. (2006). Risk factors for the transmission of hepatitis C virus infection in the Turkish population. Dig. Dis. Sci..

[49] Thursz M., Fontanet A. (2014). HCV transmission in industrialized countries and resource-constrained areas. Nat. Rev. Gatroenterol. Hepatol..

[50] Kotsiliti E. (2024). Hybrid APASL meeting 2024. Nat. Rev. Gastroenterol. Hepatol..

